# Driving Stress Detection Using Multimodal Convolutional Neural Networks with Nonlinear Representation of Short-Term Physiological Signals

**DOI:** 10.3390/s21072381

**Published:** 2021-03-30

**Authors:** Jaewon Lee, Hyeonjeong Lee, Miyoung Shin

**Affiliations:** Bio-Intelligence & Data Mining Laboratory, School of Electronic and Electrical Engineering, Kyungpook National University, Daegu 41566, Korea; realjaewon94@gmail.com (J.L.); leehj1224k@gmail.com (H.L.)

**Keywords:** stress detection, physiological signals, galvanic skin response (GSR), heart rate (HR), recurrence plot (RP), deep learning, convolutional neural network (CNN)

## Abstract

Mental stress can lead to traffic accidents by reducing a driver’s concentration or increasing fatigue while driving. In recent years, demand for methods to detect drivers’ stress in advance to prevent dangerous situations increased. Thus, we propose a novel method for detecting driving stress using nonlinear representations of short-term (30 s or less) physiological signals for multimodal convolutional neural networks (CNNs). Specifically, from hand/foot galvanic skin response (HGSR, FGSR) and heart rate (HR) short-term input signals, first, we generate corresponding two-dimensional nonlinear representations called continuous recurrence plots (Cont-RPs). Second, from the Cont-RPs, we use multimodal CNNs to automatically extract FGSR, HGSR, and HR signal representative features that can effectively differentiate between stressed and relaxed states. Lastly, we concatenate the three extracted features into one integrated representation vector, which we feed to a fully connected layer to perform classification. For the evaluation, we use a public stress dataset collected from actual driving environments. Experimental results show that the proposed method demonstrates superior performance for 30-s signals, with an overall accuracy of 95.67%, an approximately 2.5–3% improvement compared with that of previous works. Additionally, for 10-s signals, the proposed method achieves 92.33% classification accuracy, which is similar to or better than the performance of other methods using long-term signals (over 100 s).

## 1. Introduction

Excessive mental stress can negatively affect people in numerous ways, such as by causing various diseases or reducing concentration and work efficiency [1,2,3,4]. Particularly, in driving situations, stress is closely related to driving safety. For example, stress can lead to traffic accidents by impairing driving performance or reducing a driver’s ability to make decisions to cope with dangerous situations [5,6]. Thus, the problem of recognizing stress early has been tackled in several studies to reduce the possibility of traffic accidents [7,8,9].

To detect drivers’ stress, a variety of measurements have been used which can be classified into three categories, namely, vehicle motion measurements, facial behavior measurements, and physiological measurements. Vehicle motion measurements mainly include drivers’ acceleration, braking, lane position, steering angle, and handle movement patterns [10,11,12]. Such measurements are easily obtainable but dependent on vehicle types, driving habits, or road conditions. Similarly, facial behavior measurements, such as eye gaze status, pupil dilation, blink rate, yawning, and head movement, can be acquired easily without interfering with the driver [13,14,15]. However, these measurements tend to be unstable under certain conditions, such as poor lighting, bad weather, at night, or when a driver is wearing eyeglasses.

Meanwhile, physiological measurements are not affected by external factors unrelated to stress, such as lighting conditions or driving manner. Moreover, physiological signals collected by equipment attached to the body can provide relevant information on a driver’s internal state, which are effective for stress recognition [16,17,18]. As stress response is related to autonomic nervous system activity, galvanic skin response (GSR) signals associated with sweat gland activity and heart rate (HR) associated with cardiac activity are often used as reliable stress indicators [19,20]. Thus, measuring and utilizing various physiological signals from low-cost and widely available equipment is highlighted in stress recognition problems [14,21,22].

In stress recognition studies, feature extraction has been performed mainly in the time or frequency domain of physiological signals. Time domain features were typically extracted from time series segments truncated by window sliding strategies [2,6,23,24,25,26,27,28], whereas frequency domain features were extracted from low- and/or high-frequency regions [6,25,26,27,29,30]. Based on these features, statistical measures such as mean, standard deviation, skewness and kurtosis were commonly calculated and used to differentiate between stressed and non-stressed conditions [24]. Meanwhile, in other studies [14,31,32], domain-dependent features defined according to experts’ domain knowledge on specific signal types or human mental stress were used. Although such features are effective, they are generally not robust to certain variations, such as noise and intrapersonal variability.

In addition, nonlinear features, such as recurrence quantification analysis (RQA) [33] measurements, were employed in some stress recognition studies [6,34,35]. RQA measurements are to quantify the structure of a recurrence plot (RP) [36] representing the recurrence properties of time series data presented in the phase space trajectory. Although used in several studies, RQA measurements are limited in their usefulness because they provide less information than RPs themselves. So, it seems to be a prospective approach to learn and extract features directly from the RP itself. Table 1 provides the types of features that were used primarily in stress recognition studies.

Furthermore, in driving situations, short-term monitoring is essential to driving safety. However, many previous studies on stress detection used relatively long-term physiological signals, typically several minutes long [30,37]. In some recent studies [14,26,38], short-term ECG signals were often used which have high sampling frequency and correlation with stress conditions. However, ECGs are inconvenient and suffer from noise vulnerability from unstable contacts. Nowadays the leveraging of short-term (i.e., tens of seconds long) GSR or HR signals is increasing, but still knowledge-based feature engineering accompanied with conventional machine learning classifiers requires substantial time and human efforts [2,6,14,28].

To address above issues, in this study, we investigate the stress detection problem using short-term GSR and HR signals easily obtainable from wearable devices. Unlike conventional studies that utilize statistical features or domain knowledge-based feature engineering, we explored the nonlinear features presented in continuous RPs of short-term foot GSR (FGSR), hand GSR (HGSR), and HR signals, along with multimodal CNNs.

The main contributions of this study can be summarized as follows:

- Employing short-term (30-s or less) FGSR, HGSR, and HR signals, which have not been fully utilized in previous stress classification studies.
- Investigating continuous RPs (Cont-RPs) obtained by converting one-dimensional time series into two-dimensional matrices for exploring features differentiating between stressed and relaxed states.
- Proposing a multimodal CNN classifier based on Cont-RPs and validating its effectiveness in drivers’ stress classification.

## 2. Materials and Methods

**Figure 1:**
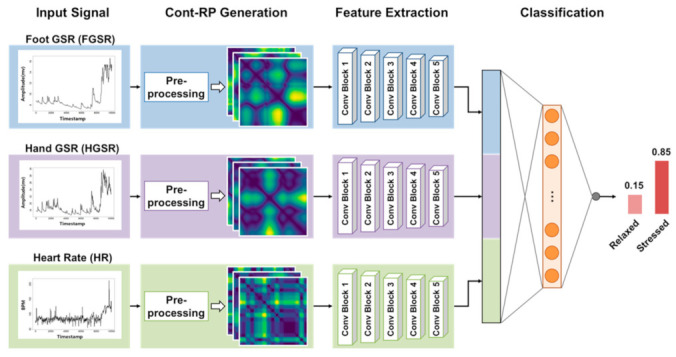
Overview of proposed multimodal CNN approach using FGSR, HGSR, and HR signals for stress class prediction.

The proposed method using multimodal CNNs for stress detection is summarized in Figure 1. The method consists of three CNNs for handling foot GSR (FGSR), hand GSR (HGSR), and HR signals. Each CNN receives a nonlinear representation of the input signals to train the network, in which the nonlinear representation is obtained by generating continuous RPs (Cont-RPs), which is explained in Section 2.3. Next, the three outputs of the last convolution block of each CNN are flattened and concatenated into one representation vector, which is fed to a fully connected layer for stress class prediction.

### 2.1. Driving Stress Dataset

The dataset used in this study was obtained from the Stress Recognition in Automobile Drivers (SRAD) dataset in PhysioNet [37]. The SRAD dataset was originally collected to determine drivers’ relative stress level based on physiological signals in real-world driving environments. The dataset contains multiple types of physiological signals related to different driving stress conditions obtained from nine healthy subjects in the context of driving a given route through open roads. The prescribed route has two rest periods before and after driving, two highway driving periods, and three city driving periods. According to [37], physiological signals from the rest and highway and city driving periods are related to low, medium, and high levels of stress, respectively.

**Figure 2:**
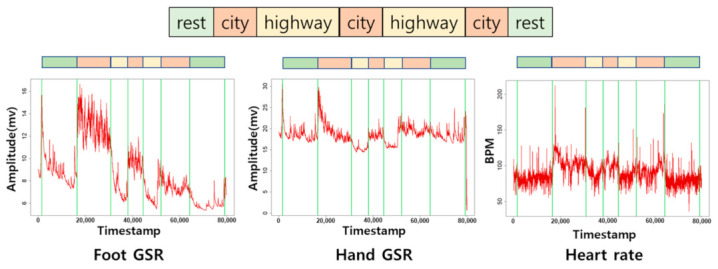
Example of multiple physiological signals within one recording segmented based on different road conditions.

A total of 17 recordings ranging from 60 to 90 min are available to the public. Each recording consists of six different physiological signals, including ECG, electromyogram (EMG), FGSR, HGSR, HR, and respiration (RESP). In addition, another time series called a “marker” is given for each recording to indicate the interval (i.e., start and end points) of each period and its corresponding stress level. Figure 2 presents an example of the three signals in one recording divided into seven segments according to the period-switching lines extracted from the marker data. The magnitude of the physiological signals clearly varied with the road conditions, which generated different stress levels.

In this study, only nine recordings (i.e., drive 06, 07, 08, 09, 10, 11, 12, 15, and 16) were used where the marker signals were clear, and all three signals were provided. The excluded eight recordings do not contain all three sensor signals, or their marker signals are not clear in terms of transient time between each period, as shown in Table 2.

The statistical characteristics of the nine recordings used in our experiment are given in Table 3. In this table, it is observed that the values of the three signals (FGSR, HGSR, and HR) are in very different ranges depending on subjects. Moreover, in some subjects, the range of signal values very overlaps between low and medium stress levels or between medium and high stress levels. To clarify the usefulness of Cont-RPs for the three signals, this study focused on the problem of distinguishing between low stress levels in rest and high stress levels during city driving.

### 2.2. Preprocessing

To analyze the three signals, namely, FGSR, HGSR, and HR, from the input recordings, we preprocessed them in the following way. First, we resampled each signal to equalize the sampling frequency and produced RPs with an appropriate size, that is, neither too large nor too small, to adequately represent time-varying patterns. Specifically, in the SRAD dataset, two types of GSR signals (i.e., FGSR and HGSR) are given at a sampling rate between 33 Hz and 35 Hz, and the HR signals have a sampling rate between 0.5 Hz and 1 Hz. Therefore, we downsampled the GSR signals and upsampled the HR signals to equally obtain 16 samples per second. By doing so, reducing computational complexity while maintaining key information on the signal changes caused by driving stress was possible.

Second, we applied a median filter to eliminate noise and artifacts that can be caused by poor electrode contact or undesirable body movements. Median filtering is efficient for reducing the impact of these spiky noises, and easier to implement than other complicated filtering techniques. Variations in the magnitude of physiological signals from different people or under different environments (external factors such as temperature and humidity) exist. Thus, to compare signals of different amplitudes, we normalized them to have zero mean and unit standard deviation.

Finally, we generated short-term (10 s and 30 s) samples by segmenting the signals into a fixed window size with a 50% overlap between adjacent segments and assigned the same label to each sample as the original. To formulate the stress prediction problem, we used only the samples labeled as one of two stress levels, in which low-level stress (i.e., relaxed) was assigned to the rest periods, and high-level stress (i.e., stressed) was assigned to the city driving periods. If some transitions are involved in one sample, from the relaxed state to the stressed state, or vice versa, the sample was not considered for further analyses.

### 2.3. Stress-Relevant Characteristics of Cont-RPs

To investigate nonlinear properties distinct in the stressed and relaxed states, we converted the one-dimensional time series into two-dimensional matrices representing Cont-RPs. A conventional RP [36] is represented as a binary matrix, indicating the recurring states of dynamic system in the phase space. Specifically, we marked each component in the RP matrix as 1 if the distance between the two states in the phase space is less than the predefined threshold value, and 0 otherwise. Existing RP-based studies are based mainly on these binarized RPs.

Unlike such studies, we attempted to utilize all the information contained in the matrix of distances between the two states in the phase space without binarization. Therefore, the RP matrix used in this study contained components with continuous values, indicating the degree of recurrence between the two states, rather than binary values, which are referred to as Cont-RPs.

**Figure 3:**
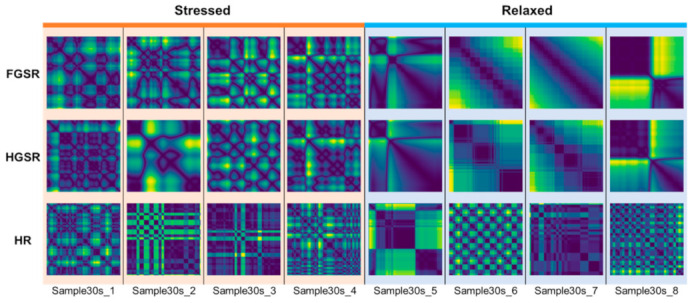
Examples of Cont-RPs for short-term (30 s) FGSR, HGSR, and HR signals.

**Figure 4:**
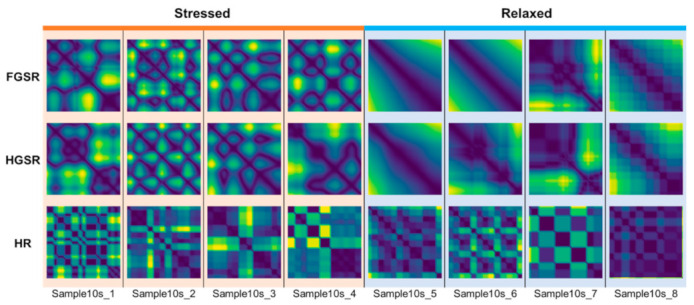
Examples of Cont-RPs for short-term (10 s) FGSR, HGSR, and HR signals.

Examples of Cont-RPs for the three short-term FGSR, HGSR, and HR signals are presented in Figure 3 (for 10 s) and Figure 4 (for 30 s). We set the embedding dimension to 3 and the time delay to 2 so that the resulting Cont-RPs can well classify between stressed and relaxed states. In the figures, the magnitude of the components in the Cont-RPs is the Euclidean distance between two embedded states in phase space, visualized as color intensities with low and high values shown as dark blue and yellow, respectively.

Overall, the Cont-RP patterns showed the difference between the stressed and relaxed states. One distinct trait was that complex and irregular patterns were observed in the stressed state, whereas monotonous and regular patterns were observed in the relaxed states. Specifically, clearer distinctions between the two states were observed in the examples of both GSR signals regardless of their length (both 30 s and 10 s). Netting or bubble-shaped patterns appeared irregularly in the stressed conditions, whereas smooth transitions were mostly observed in the relaxed state, with few drastic transitions in intensity changes. In addition, slight differences were observed between the FGSR and HGSR signals, despite both being GSRs. This outcome appeared presumably because the two GSR signals were measured on drivers’ different body parts doing different activities while driving. Thus, they are likely complementary when used together.

Meanwhile, the Cont-RPs of the HR signals had characteristics differing from those of the Cont-RPs of the GSR signals. Figure 3 and Figure 4 show checkerboard patterns with regularly arranged squares with a similar size in the relaxed state, thereby indicating that the HR signals changed regularly. Meanwhile, in the stressful situations, bright vertical and horizontal lines appeared irregularly in contrast to the relaxed state. In addition, given the short-length (10 s) of the signals, their Cont-RPs manifested more localized patterns that corresponded to the 1/9 region of the 30 s Cont-RPs, as shown in Figure 3 and Figure 4.

The Cont-RPs of the three types of signals exhibited different aspects of characteristics related to stress. Therefore, examining the three signals individually to extract stress-relevant features would be worthwhile. For this purpose, we employed multimodal CNN, which is detailed below.

### 2.4. Feature Learning and Classification Based on Cont-RPs

**Figure 5:**
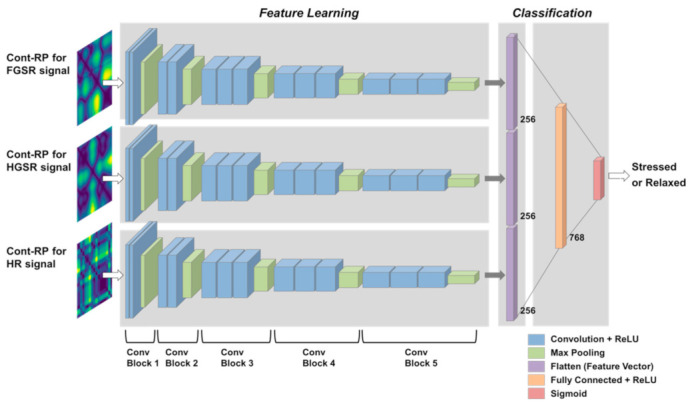
Detailed configuration of proposed multimodal CNN model for feature learning and stress classification.

To automatically learn the stress-relevant features of the three types of physiological signals, we constructed a multimodal CNN model consisting of three CNNs for the three signals and one dense layer to generate the probabilities of each stress class (e.g., stressed or related) from their outputs. As inputs for each CNN for training, the Cont-RPs of each type of signal were given. That is, three CNNs were for learning the inherent features contained in the Cont-RPs of FGSR, HGSR, and HR signals. The entire procedure is depicted in Figure 5, which can be divided into three phases, that is, first, learning representative feature maps through the three CNNs from the Cont-RPs of the three types of signals; second, combining their flattened outputs into one integrated representation vector; and third, generating class probabilities based on the representation vectors by learning the relationships between them through a fully-connected layer with a sigmoid function.

To learn the representative feature maps of the Cont-RPs, we built three CNNs with the same architecture, as depicted in Figure 5. The architecture of our CNNs is the same as the front part of the VGG16 model [39], but all the parameters were trained from scratch based on our training dataset. That is, each CNN consisted of five convolution blocks, each of which contained a two-dimensional convolution, rectified linear unit (ReLU) activation, and max pooling layer. The first two convolution blocks (i.e., ConvBlocks 1 and 2) included two convolution layers, whereas the remaining blocks contained three convolution layers. In addition, 3 × 3 filters with a stride of 1 were used for all the convolution layers and 2 × 2 filters with a stride of 2 were used for the max pooling layer, similar to [39]. The convolution layers learn spatial information and extract features using kernels with sliding window strategy, while pooling layers reduce the spatial dimension of feature maps. After going through the fifth convolution block in each CNN, the resulting three feature maps were flattened (i.e., three vectors with dimension of 256) and combined into one integrated representation vector with dimension of 768. Subsequently, this vector was fed to the dense layer, and the sigmoid function was used as the activation function for the output layer to predict the probabilities of the two classes, that is, the stressed and relaxed states.

## 3. Results and Discussion

### 3.1. Experimental Setup

For the experiments, we implemented the proposed method in Python using Keras with Tensorflow backend and carried out with an NVIDIA Titan X and 12 GB of RAM. In the SRAD dataset, the number of samples for the relaxed state (minority class) was fewer than the number of samples for the stressed state (majority class). The classifier from the imbalanced data was prone to bias toward the majority class, whereas the minority class samples were not well learned. To handle the class imbalance problem, we performed random undersampling on the stressed samples to balance the class distribution between the stressed and relaxed states. Consequently, we obtained 1872 30-s samples and 5348 10-s samples for the model development.

To assess the effectiveness of the proposed method, we used leave-one recording-out (i.e., recording-wise) cross-validation. That is, for each round, we used one recording for testing and the remaining eight recordings for model training. We conducted this procedure to evaluate the generalization ability of the trained model for new recordings of completely unknown individuals not included in the training data. We performed a grid search to get optimal hyperparameters such as learning rate, batch size and the number of epochs. For example, we considered a suite of different batch sizes from 1 to 20, the number of epochs between 1 to 30, and learning rate from 0.1 to 0.00001. We then trained our multimodal CNN model for 15 epochs with a batch size of 4 and used the Stochastic Gradient Descent (SGD) optimizer with a learning rate of 0.001 for the weight update.

We measured classification performance using accuracy, precision (positive predictive values), recall (sensitivity), the F1-score, and area under the curve (AUC). Accuracy and the AUC describe the overall performance across all classes of samples, whereas precision, recall and the F1-score calculated for each class present a method’s ability to distinguish between certain classes. These indices can be calculated as follows:

Accuracy = (TP + TN)/(TP + TN + FP + FN) × 100%,

Precision = TP/(TP + FP) × 100%,

Recall = TP/(TP + FN) × 100%,

F1-score = (2 × Precision × Recall)/(Precision + Recall)

where TP is the number of correctly classified positive samples, TN is the number of correctly classified negative samples, FP is the number of negative samples incorrectly classified as positive and FN is the number of positive samples incorrectly classified as negative.

### 3.2. Performance Evaluation

We evaluated the performance of the proposed stress detection approach using two different lengths (i.e., 10-s and 30-s) of input signals. The overall classification results are summarized in Table 4. From this table, it is found that for the 30-s input signals, overall classification accuracy (and F1-score) was 95.67%, whereas that for the 10-s input signals was 92.33%. Moreover, the AUC difference between the 30-s and 10-s input signals was not exceedingly large, with the AUC as 0.9870 and 0.9619, respectively.

**Figure 6:**
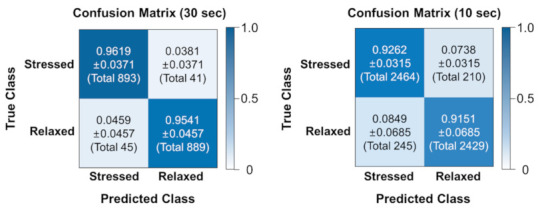
Aggregated confusion matrices for each input signal length (30 s and 10 s). The number in parentheses of each quadrants of the confusion matrices indicates the total number of samples classified as each case.

Figure 6 represents aggregated confusion matrices which include the aggregation of all classification results obtained from leave-one recording-out cross-validation. We can observe that only a small portion of stressed and relaxed samples (3.8% of stressed samples and 4.6% of relaxed samples for classification using 30-s signals; 7.4% of stressed samples and 8.5% of relaxed samples for classification using 10-s signals) were incorrectly classified into each other.

Interestingly, reducing the length of the input signals from 30 s to 10 s did not significantly degrade our proposed method’s ability to distinguish between the stressed and relaxed states. This finding showed that our proposed method performed relatively well in detecting the stressed state using only short-length 10-s inputs.

**Figure 7:**
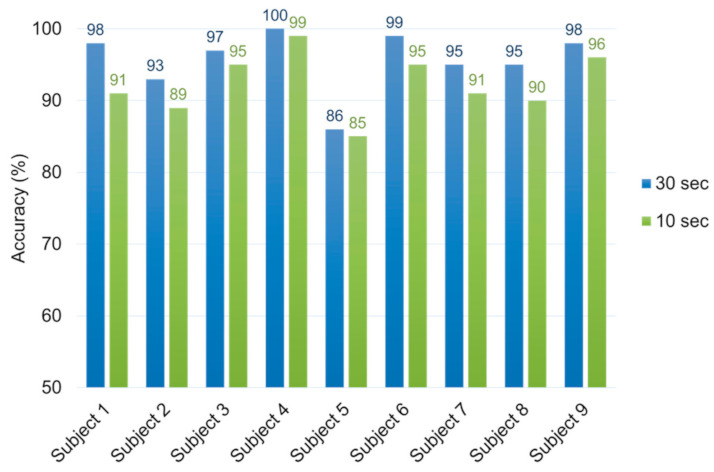
Classification performance in individual recordings based on input signal length (30 s and 10 s).

As shown in Figure 7, the proposed method’s classification performance in the individual recordings varied from 86% to 100% for the 30-s signals and from 85% to 99% for the 10-s signals. In addition, when using the 10-s signals, classification accuracy decreased slightly by 1 to 7% compared with when using the 30-s signals.

Next, we compared the performance of our multimodal CNN model with that of three unimodal CNNs for the FGSR, HGSR, and HR signals, and the results are presented in Table 5. This table shows that the combined use of the three physiological signals significantly improved classification performance by approximately 5% to 33% for the 30-s signals and by 2% to 36% for the 10-s signals. Moreover, among the three unimodal CNNs, the FGSR-CNN demonstrated a fairly satisfactory performance, whereas the HR-CNN exhibited a fairly poor performance. The accuracy of the HGSR-CNN decreased by approximately 10% to 14% depending on the length of the input signals compared with that of the FGSR-CNN.

Regarding the variations in model performance, there was not much difference between 30-s and 10-s signals in FGSR. That is, the performance of stress detection with FGSR was not significantly affected by the length of input signal. This indicates that the Cont-RPs of short-term FGSR signals can be effective for stress classification in that they will reflect the traits able to differentiate between stressed and relaxed states. Moreover, HGSR performs slightly better for 10-s signals than for 30-s signals, which means that the short-term HGSR signal may be a more reliable indicator for stress detection than the long-term HGSR signal.

**Figure 8:**
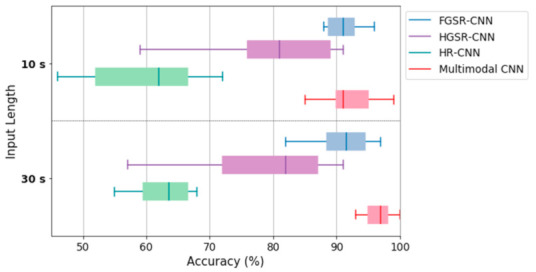
Comparison of the performance variations of our multimodal CNN model and three unimodal CNNs for FGSR, HGSR and HR signals depending on input signal length and sensor type.

Further, the change in performance among different recordings is not very significant in our multimodal CNN model for both 30-s and 10-s signals, compared to other unimodal CNNs (refer to Figure 8). Of the three unimodal CNNs, FGSR is the most stable for different recordings regardless of the input signal length. For HR, longer signals appear more stable when classifying stressed/relaxed states. That is, 30-s signals have less variations between different recordings than 10-s signals.

We also compared the classification performance of multimodal CNN with two baseline neural network models. All three models in Table 6 used CNN architecture to extract features from FGSR, HGSR, and HR signals, and perform stress classification via concatenation of three vectors and fully connected layers as proposed multimodal CNN. Specifically, the multimodal 1-D CNN is a classifier that takes three one-dimensional sequence of FGSR, HGSR, and HR, and outputs the probability of stressed and relaxed states. The three inputs are the preprocessed signals before generating Cont-RPs. The multimodal VGG16 model has the same structure as proposed multimodal CNN but uses default parameters of existing VGG16 without parameter learning.

Table 6 shows that the performance of two models (multimodal CNN and multimodal VGG16) with three Cont-RPs as input is approximately 1% to 12% and 1% to 9% higher than that of multimodal 1-D CNN model with one-dimensional sequence as input for 30-s and 10-s input signal, respectively. The results indicate that Cont-RPs have more useful features for stress classification than one-dimensional signals as itself which have been used in most previous studies. Even with the same VGG structure, the performance of multimodal CNN model which have learned stress-relevant features based on Cont-RP samples is about 8% to 10% higher than that of multimodal VGG16 model.

### 3.3. Comparison with Related Works

A number of related studies were conducted on the problem of classifying drivers’ stress based on physiological signals, including GSR and/or HR. Table 7 shows the classification performance of other two-class (stressed and relaxed) stress classification methods compared with that of the proposed method. Although some of the other methods were tested using different datasets, they were also included in this table for reference.

Most of the studies in this table used multisensor signals for detecting drivers’ stress, which is favorable in the creation of a reliable system. For example, Wang et al. [40] proposed a CNN-based driving stress detection scheme using GSR, HR, HRV and breathe rate, and obtained 92% accuracy in a two-class classification. It is worth noting that the authors analyzed their own collected dataset, which differs from the dataset (i.e., SRAD) used in our experiments. Jiménez-Limas et al. [30] detected two driver stress classes using logistic regression and 5-min FGSR and HR signals and respiration rate. As drivers’ conditions should be detected as quickly as possible to prevent potential accidents, long signals (over 100 s) are not very helpful in detecting drivers’ stress early in actual situations. Lopez-Martinez et al. [41] classified two stress levels using support vector machine (SVM) and achieved high accuracy. However, the achieved performance is 2.67% lower than that of our model based on the same length (30 s) of signals.

In addition to Table 7, where comparison was done with the performance of stress classification for two classes, there are other studies using the same SRAD dataset to perform stress level classification for three classes which is slightly different from our purpose. Chen et al. [26] extracted features from GSR, ECG and respiration data and achieved 89.7% accuracy in by kernel-based classifiers. Meanwhile, Wang et al. [42] employed Adaboost classifier for driving stress detection system by using only FGSR data and resulted in high accuracy (90.09%). Although a single sensor is convenient to use, unstable results can occur in that no additional sensor data compensate for lost information due to inaccurate and missing signals. Healey and Picard [37] classified 5 min intervals of data into three stress levels with linear discriminant analysis (LDA). They used ECG, EMG, respiration rate and two GSR data, achieving the highest accuracy in the listed studies. Singh et al. [43] utilized 10 s signals of HGSR and photoplethysmogram (PPG) sensor for three-class classification, resulted in 89.23% accuracy. It is worth noting that they analyzed their own collected dataset, which is different from the data we used (SRAD) in our experiments.

The significance of our proposed method is in employing short-term (30-s or less) signals of GSR and HR, which are acquired while real driving but not fully utilized in stress classification. As mentioned in Section 1, considering GSR and HR related to ANS activity, experimental results have shown that GSR and HR signals of short length (30-s or less) are useful as stress indicators. Although the proposed model achieved good performance for unseen recording in leave-one recording-out cross-validation, the performance of our model is still limited by the small number of samples used in the experiment due to the lack of public available dataset collected in the actual driving environment. In the future, we plan to measure more physiological signals in simulated driving environment and conduct additional experiments to obtain more reliable results for the proposed model.

### 3.4. Visualization of Learned Feature Distributions

**Figure 9:**
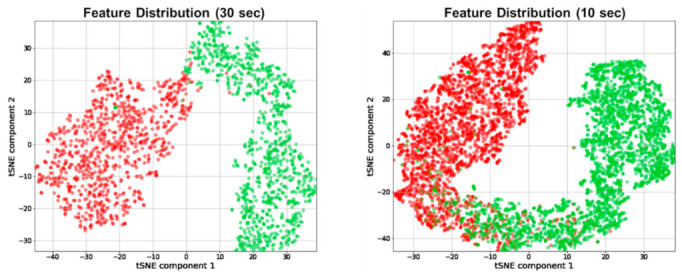
Distribution of our learned representation vectors embedded in two-dimensional vector space learned from FGSR, HGSR, and HR signals (red: stressed, green: relaxed).

As mentioned in Section 2.4, a set of convolution blocks (1~5) in each CNN extracted features from the Cont-RPs of each type of input signal and outputted a 256-dimensional vector. As we employed three types of physiological signals as input, the concatenation of the three outputs led to one 253 × 3 = 768 integrated representation vector, which was then fed to a fully connected layer for stress classification. To visually confirm that the representation vectors learned from the Cont-RPs of the FGSR, HGSR, and HR signals were discriminative between classes, we reduced the dimension of the representation vectors from 768 to 2 using the *t*-stochastic neighbor embedding (*t*-SNE) method [44] and visualized them in a reduced two-dimensional space, as shown in Figure 9. The *t*-SNE method is a nonlinear dimensionality reduction technique that can transform high-dimensional vectors into low-dimensional vectors by preserving the intrinsic structure of the data.

In Figure 9, the representation vectors are colored by class, with red and green denoting the stressed and relaxed classes, respectively. The two classes were well distinguished in terms of 30-s and 10-s signals, which illustrated that our model learned satisfactory features from the Cont-RPs of the FGSR, HGSR, and HR signals for distinguishing between classes.

Interestingly, Figure 9 shows that the distributions of the two classes were in the form of their own long bands. Given that we split the continuous long-time signals into multiple short-length segments, we can infer that the learned representation vectors not only distinguish between classes but also contain consecutive temporal features between them.

## 4. Conclusions

In this study, we proposed a new method for detecting drivers’ stress based on short-term physiological signals, namely, FGSR, HGSR, and HR, which can be easily obtained by wearable devices. Specifically, by constructing the two-dimensional nonlinear representation of the Cont-RPs of short-term (10 s and 30 s) input signals, we were able to learn their corresponding satisfactory representation vectors through multimodal CNNs that can well distinguish between stressed and relaxed states. Experimental results using the publicly available SRAD dataset showed that the proposed method demonstrated superior performance in detecting drivers’ stress, overall accuracy of 95.67% with 30-s signals and 92.33% with 10-s signals, and a performance improvement of approximately 2.5–3% compared with conventional studies using long-term (100 s or more) signals.

As demand to leverage various types of physiological signals acquired by wearable devices increases, the proposed method is expected to be widely used for detecting drivers’ stress in real driving scenarios. Moreover, the proposed method’s use of short-term signals may be highly attractive for real-time applications. In future research, we plan to determine whether the proposed method can be applied to other types of physiological signals.

## Figures and Tables

**Table 1:** Type of features often used in stress recognition studies using physiological signals.

Feature Domain	Physiological Signals	Feature Examples	Study
Time	GSR, ECG, HR, ST, BR, SpO2, BVP	Mean, Median, SD, RMS, Skewness, Kurtosis, Maximum, Minimum, Interquartile range, Sum, Amplitude, Rise time, Means of differences between adjacent elements, Number of peaks	[2,6,23,24,25,26,27,28]
Frequency	GSR, ECG, RSP	Entropy, Power spectrum density, Power sum, The average power, LF, HF, Ratio of LF/HF, Spectral peak features	[6,25,26,27,29,30]
Domain-dependent	GSR, ECG, RSP, EMG	Mean HP, Variation in HP, Variation in GSR, Differential area between GSR and its first-order interpolation, Product between RMS and SDCC, Trend-based feature generation	[14,31,32]
Nonlinear	ECG	RP, RQA, Poincare plot	[6,34,35]

GSR: galvanic skin response; ECG: electrocardiogram; HR: heart rate; ST: skin temperature; BR: breath-flow rate; SpO2: oximetry; BVP: blood volume pressure; RSP: respiration; EMG: electromyogram; SD: standard deviation; RMS: root mean squares; LF: low frequency; HF: high frequency; HP: heart period; SDCC: standard deviation of the frequencies; RP: recurrence plot.

**Table 2:** Excluded recordings in our paper and the reasons.

Excluded Recording	Reason
drive 01	Marker signal is missing.
drive 02	HGSR signal is missing.
drive 03	Marker and HR signals are missing.
drive 04	Marker signal is not clear.
drive 05	HR signal is missing.
drive 13	HGSR signal is missing.
drive 14	HR signal is missing.
drive 17	Marker signal is missing.

**Table 3:** Mean and standard deviation of the three physiological signals for 9 recordings used in experiment.

Sensor		FGSR			HGSR			HR	
Status (Stress Level)	Rest (Low)	Highway Driving (Medium)	City Driving (High)	Rest (Low)	Highway Driving (Medium)	City Driving (High)	Rest (Low)	Highway Driving (Medium)	City Driving (High)
drive 06	7.42 ± 1.80	7.25 ± 1.22	10.29 ± 2.64	18.36 ± 1.32	16.19 ± 1.77	19.36 ± 1.91	80.24 ± 9.35	88.31 ± 10.50	99.75 ± 13.19
drive 07	9.21 ± 3.36	12.76 ± 1.16	12.81 ± 1.72	5.46 ± 1.71	6.76 ± 1.17	7.75 ± 1.20	70.9 ± 8.41	73.44 ± 5.55	78.22 ± 7.60
drive 08	2.89 ± 0.93	6.44 ± 0.90	6.80 ± 1.19	3.21 ± 0.67	5.45 ± 0.97	6.03 ± 1.54	63.65 ± 12.53	66.49 ± 11.04	74.87 ± 24.93
drive 09	3.55 ± 1.70	5.12 ± 0.99	5.27 ± 1.10	4.40 ± 2.39	5.66 ± 1.35	6.60 ± 1.69	71.24 ± 15.33	73.36 ± 18.20	74.03 ± 15.36
drive 10	4.62 ± 3.23	6.96 ± 2.12	9.66 ± 2.23	6.98 ± 4.05	6.44 ± 1.75	9.32 ± 2.60	75.35 ± 10.60	77.66 ± 7.92	83.73 ± 12.99
drive 11	3.24 ± 0.89	5.61 ± 0.86	6.23 ± 1.28	3.53 ± 1.21	7.32 ± 1.36	8.52 ± 1.94	60.64 ± 9.53	71.42 ± 21.00	75.54 ± 23.85
drive 12	3.32 ± 2.99	4.07 ± 1.27	5.35 ± 3.40	7.67 ± 2.70	15.44 ± 2.21	15.53 ± 2.00	78.72 ± 4.57	87.59 ± 4.06	88.44 ± 6.32
drive 15	4.35 ± 1.38	6.84 ± 0.80	7.69 ± 1.37	4.55 ± 1.01	6.67 ± 1.25	7.77 ± 1.86	69.83 ± 24.91	67.98 ± 11.01	72.36 ± 14.48
drive 16	3.74 ± 0.91	5.71 ± 0.74	6.90 ± 1.31	16.09 ± 1.84	20.10 ± 1.07	21.21 ± 2.11	89.16 ± 10.30	101.9 ± 12.65	106.1 ± 17.57

**Table 4:** Classification performance of proposed method based on input signal length.

Input Length	Class	Precision (PPV)	Recall (Sensitivity)	F1-Score	Overall Accuracy	AUC
30 s	Stressed	95.7%	96.0%	95.8%		
	Relaxed	95.9%	95.8%	95.7%		
		95.89%	95.67%	95.67%	95.67%	0.9870
10 s	Stressed	91.7%	92.8%	92.3%		
	Relaxed	92.4%	91.7%	91.9%		
		91.67%	92.78%	92.33%	92.33%	0.9619

**Table 5:** Classification performance of proposed method based on input signal length and sensor type.

Signal		Stressed		Relaxed		Overall		
Length	Type	Precision	Recall	Precision	Recall	F1-Score	Accuracy	AUC
30 s	FGSR	92.67%	87.50%	89.67%	92.50%	90.62%	90.83%	0.9091
	HGSR	82.71%	79.57%	82.86%	77.00%	76.57%	78.29%	0.7825
	HR	67.25%	59.75%	64.25%	66.00%	61.00%	62.50%	0.6274
	3 types	95.67%	96.00%	95.89%	95.78%	95.67%	95.67%	0.9870
10 s	FGSR	92.88%	88.50%	89.63%	92.38%	90.50%	90.38%	0.9101
	HGSR	83.56%	82.67%	83.56%	79.00%	79.83%	80.67%	0.8141
	HR	63.86%	61.86%	55.57%	57.43%	56.71%	59.57%	0.5963
	3 types	91.7%	92.8%	92.4%	91.7%	92.33%	92.33%	0.9619

**Table 6:** Classification performance of proposed method compared with baseline CNN classifier.

Signal	Input	Classification	Stressed		Relaxed		Overall
Length	Type	Model	Precision	Recall	Precision	Recall	Accuracy
30 s	1-D sequence	Multimodal 1-D CNN	82.56%	86.78%	86.89%	80.22%	83.44%
	Cont-RP	Multimodal VGG16	87.88%	81.88%	85.22%	86.11%	84.11%
	Cont-RP	Multimodal CNN	95.67%	96.00%	95.89%	95.78%	95.67%
10 s	1-D sequence	Multimodal 1-D CNN	83.11%	84.33%	86.33%	82.44%	83.33%
	Cont-RP	Multimodal VGG16	84.55%	81.33%	84.55%	86.44%	84.00%
	Cont-RP	Multimodal CNN	91.7%	92.8%	92.4%	91.7%	92.33%

**Table 7:** Comparison of other 2-class stress classification methods in real-time driving scenarios.

Method	Dataset	Used Signals	Input Length	Classifier	Accuracy
[30]	SRAD	FGSR, HR, RESP	5 min	Logistic Regression	81.39%
[40]	Self-collection	HGSR, HR, HRV, Breath Rate	100 s	CNN	92%
[41]	SRAD	FGSR, HGSR, HR	30 s	SVM	93%
Proposed	SRAD	FGSR, HGSR, HR	30 s	Multimodal CNN	95.67%
Proposed	SRAD	FGSR, HGSR, HR	10 s	Multimodal CNN	92.33%

HRV: heart rate variability; RESP: respiration.
